# Research of a Novel Ag Temperature Sensor Based on Fabric Substrate Fabricated by Magnetron Sputtering

**DOI:** 10.3390/ma14206014

**Published:** 2021-10-12

**Authors:** Zong-Yao Yan, Jian-Yong Liu, Jia-Rong Niu

**Affiliations:** 1 School of Textile Science and Engineering, Tiangong University, Tianjin 300387, China; yanzongyao96@163.com; 2 Key Laboratory of Advanced Textile Composites Ministry of Education, Tiangong University, Tianjin 300387, China

**Keywords:** flexible temperature sensor, coated polyester fabric, magnetron sputtering, Ag layer

## Abstract

TPU-coated polyester fabric was used as the substrate of a flexible temperature sensor and Ag nanoparticles were deposited on its surface as the temperature sensing layer by the magnetron sputtering method. The effects of sputtering powers and heat treatment on properties of the sensing layers, such as the temperature coefficient of resistance (TCR), linearity, hysteresis, drift, reliability, and bending resistance, were mainly studied. The results showed that the TCR (0.00234 °C^−1^) was the highest when sputtering power was 90 W and sputtering pressure was 0.8 Pa. The crystallinity of Ag particles would improve, as the TCR was improved to 0.00262 °C^−1^ under heat treatment condition at 160°. The Ag layer obtained excellent linearity, lower hysteresis and drift value, as well as good reliability and bending resistance when the sputtering power was 90 W. The flexible temperature sensor based on the coated polyester fabric improved the softness and comfortableness of sensor, which can be further applied in intelligent wearable products.

## 1. Introduction

With the development of flexible, wearable [1], and portable electronic devices [2], a number of intelligent wearable products have been integrated into people’s lives [3,4,5,6]. In recent years, flexible electronic devices have shown explosive growth, and a new generation of flexible wearable sensors has become the core of intelligent flexible electronic systems. These sensors played a crucial role and have important applications in many fields, such as health monitoring [7] and medical care [8,9]. Body temperature is an essential physiological parameter of human health [10]. Disordered body temperature may predict various diseases. Since 2019, COVID-19 had become a global pandemic [11]. Fever is a crucial physiological signal of infection with the virus. So, it is particularly vital to monitor temperature continuously and accurately. Traditional temperature measurement equipment, including mercury thermometer and infrared thermometer, can only achieve measurement at a given point of time. They cannot provide real-time temperature data [12]. In order to reach continuous and stable temperature monitoring, comfortable flexible temperature sensors are needed and will be widely applied in the temperature monitoring of wearable products [13,14].

Types of flexible temperature sensors mainly include thermal resistance sensors [15], thermocouple sensors [16], and thermistor sensors [17]. Mu et al. [18] designed a thermal resistance sensor of Pt as temperature sensing layer with a serpentine structure on a polyimide substrate. Its temperature coefficient of resistance (TCR) reached 0.00204 °C^−1^ in the temperature range of 25–100 °C. Application of the thermal resistance sensor in the field of health monitoring, clinical diagnosis, and treatment is particularly beneficial. Justus et al. [19] deposited Cu on cellulose fabrics and used Al as the second thermoelectric material to construct a flexible thermocouple sensor. The experimental results showed that the thermoelectric coefficient of the sensor was 3–4 μV/K, and the output voltage was 0.4 mV at a temperature difference of 71 K. This demonstrated the potential of flexible thin-film-coated textiles as materials for the construction of sensors and their integration into garments to form functional e-textiles. Wang et al. [20] fabricated a thermistor using graphene and sodium alginate. It was deposited on non-woven fabrics to produce a flexible thermistor temperature sensor with a negative TCR. It was found that the sensor had a good TCR of −1.5 °C^−1^, and the accuracy was 0.1 °C. It can be attached to the human body and used to measure the temperature continuously and stably.

In recent years, there have been a few reports on the fabrication of flexible temperature sensors based on textile substrates by magnetron sputtering. Most researchers used polymer films as the substrate, such as polyimide [21], polyethylene terephthalate [22], and polyethylene naphthalate [23] etc. Joon et al. [24] applied the magnetron sputtering method to produce a flexible temperature sensor with Pt as the temperature sensing layer and polyimide as the substrate. It has also been reported that alloy could be used as the substrate for flexible temperature sensors. In the work of Lei et al. [25], NiCrAlY films and AlON/Al_2_O_3_ films were deposited on the Hastelloy tapes to improve the stability of the sensor. Then, Pt was deposited on top of them as the temperature sensing layer by magnetron sputtering. In fact, the flexible temperature sensors based on polymer and alloy were far from meeting the comfort requirements of wearable products. The flexibility of sensors is the key to intelligent wearable products. Integrating the temperature sensing layer into the fabric can improve the softness of sensors.

Metal nanostructures, such as nanowires (NWs) or nanoparticles, are particularly attractive to flexible and wearable electronic products because of their very high electrical conductivities [26]. Silver (Ag) is attracting increasing interest in textile applications that require electrical conductivity, antimicrobial properties, or a shiny metallic appearance for decorative purposes [27]. In this paper, silver nanoparticles were deposited on the TPU-coated polyester fabric as a temperature sensing layer by magnetron sputtering. The effects of sputtering power and heat treatment on the properties of the temperature sensing layer were studied, which laid a foundation for the fabric-based flexible temperature sensor.

## 2. Materials and Methods

### 2.1. Materials and Test Instruments

**Figure 1:**
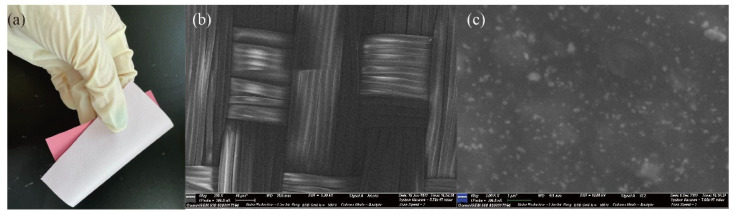
(**a**) TPU-coated polyester fabric; (**b**) SEM of uncoated fabric surface;(**c**) SEM of TPU-coated fabric surface.

The surface of the textile substrate is very unsmooth, so even under the same sputtering condition, the film structures at different positions of the textile substrate are completely different. In order to increase the smoothness of the fabric substrate and reduce the effect on resistance because of the non-smooth substrate, TPU-coated polyester fabric was used as the substrate of a flexible temperature sensor, which further increased the conductivity and stability of the resistance of the Ag temperature sensing layer. Figure 1a shows the TPU-coated polyester fabric, Figure 1b shows SEM figure of polyester fabric, and Figure 1c shows SEM figure of TPU-coated polyester fabric. It can be seen that the surface of the substrate was smoother than the uncoated polyester fabric and had no voids. The smoothness of the substrate surface is the key to the stability of sensors. Compared with uncoated polyester fabric, the conductivity and stability of the Ag temperature sensing layer deposited on TPU-coated polyester fabric were effectively improved. The electrode of the sensor was made up of 8000B-E silver paste, supplied by Shenzhen Baojiayi Technology Co., Ltd. (Shenzhen, China). The silver target material (99.99% purity) and zinc oxide target material (99.99% purity) used in the magnetron sputtering machine were supplied by Beijing Zhongcheng New Material Technology Co., Ltd. (Beijing, China).

**Figure 2:**
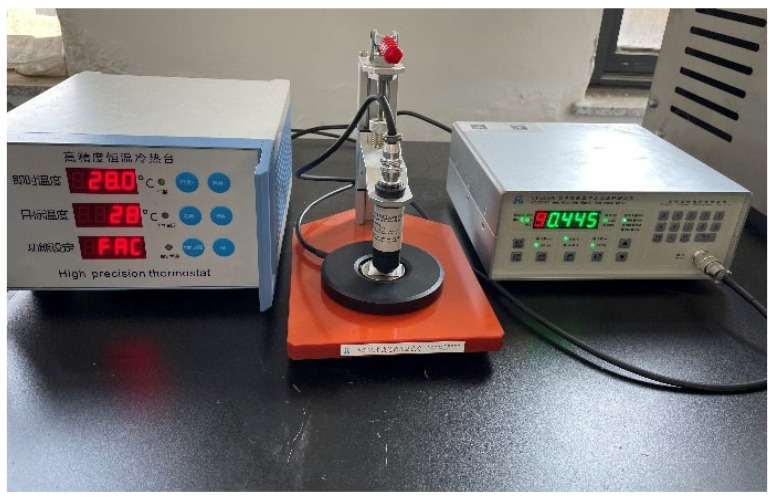
Square resistance test system.

The magnetron sputtering machine used in the experiment was supplied by Shenyang Qihui Vacuum Co., Ltd. (Shenyang, China), the ultrasonic cleaning instrument (DL-180B) for cleaning the impurities on the surface of polyester fabric by Shanghai Xinxin Instrument Co., Ltd. (Shanghai, China), and the air drying oven (GZX-GF101) by Shanghai Herde Experimental Equipment Co., Ltd. (Shanghai, China). Vacuum heat treatment were performed in a vacuum sintering furnace (GSL-1400X), supplied by Hefei Kejing Materials Technology Co., Ltd. (Hefei, China). A high-precision constant temperature table (WT-3000-12S) was used to control temperature in the experiment, supplied by Shanghai Micrograph Instrument Technology Development Co., Ltd. (Shanghai, China). A multifunctional four-probe tester (ST2258C) was used to measure the square resistance of the Ag layers, supplied by Suzhou Lattice Electronics Co., Ltd. (Suzhou, China). As shown in Figure 2, the constant temperature table was placed on the platform of four-probe tester to form a measuring system that can monitor the change of square resistance in real time, measuring the change of Ag layers square resistance between 25 and 42 °C. The crystallization of Ag particles was measured by X-ray diffraction (D8 Advance) equipment (Brook, Karlsruher, Germany). The surface morphology of Ag layers was observed by Geminisem 500 (Carl Zeiss, Jena, UK).

### 2.2. Fabrication of Flexible Ag Temperature Sensors

**Figure 3:**
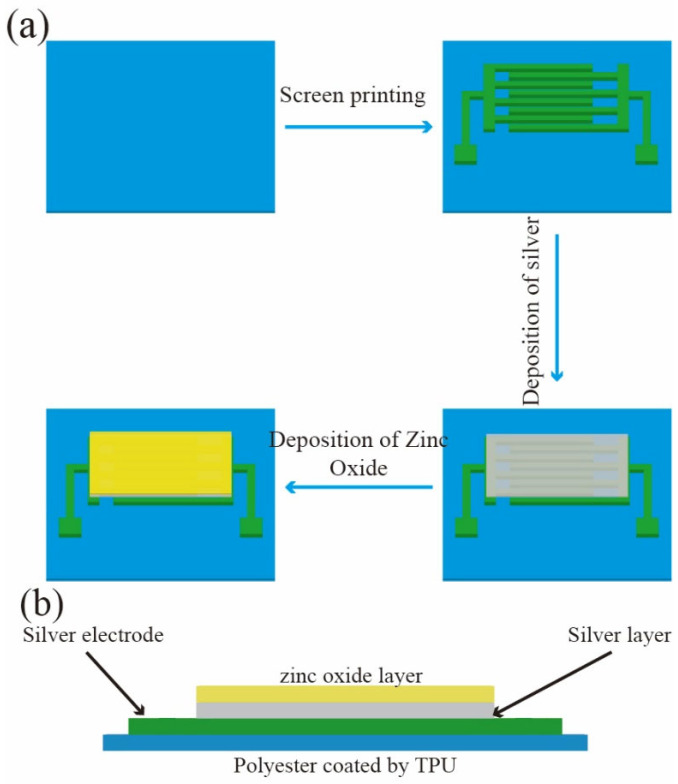

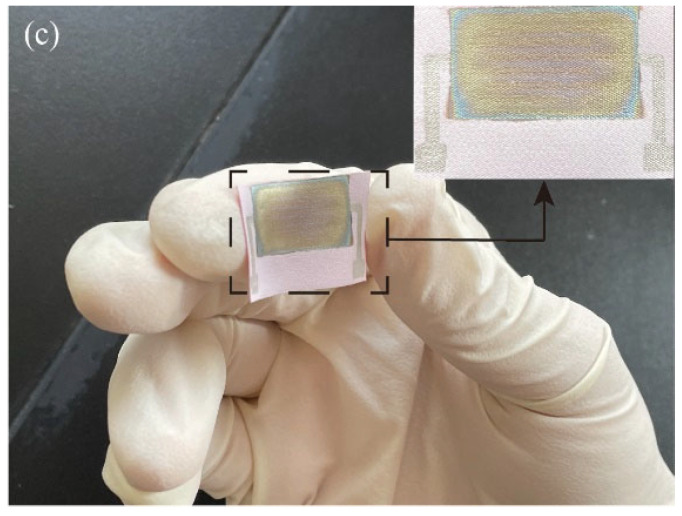
(**a**) Schematic diagram of flexible temperature sensor fabrication process; (**b**) Cross section schematic diagram of flexible temperature sensor; (**c**) Picture of flexible temperature sensor.

As shown in Figure 3a, the fabrication process of the electrode, temperature sensing layer, and isolation layer of the sensor were as follows. Firstly, the coated polyester fabric was treated with the ultrasonic cleaning instrument for 30 min to remove impurities on the surface, and then the coated polyester fabric was washed with deionized water repeatedly and dried in the oven at 60 °C. The silver paste was printed on the 10 cm × 10 cm coated polyester fabric as cross finger electrode by screen printing method. The printed fabric was put into the oven to dry at 60 °C for 20 min. A 200-nm thick Ag layer was deposited on printed substrate by magnetron sputtering. Magnetron sputtering power had a certain effect on Ag layer properties. So, in this work, eight specific sputtering powers, i.e., 50 W, 60 W, 70 W, 80 W, 90 W, 100 W, 110 W, and 120 W, were used to deposit Ag nanoparticles on the printed substrate. Although the sputtering pressure also had an effect on the deposition of Ag, considering that the deposition rate of Ag nanoparticles increases with the increment of the sputtering pressure, the sputtering pressure of 0.8 Pa was fixed in order to control the appropriate deposition rate.

In order to study the effect of heat treatment on the properties of temperature sensing layer, a set of the samples was placed in the vacuum sintering furnace for 60 min at the same sputtering conditions. Because the substrate was TPU-coated fabric, too high a heat treatment temperature would lead to fiber hurt and deformation. So, 160 °C was used as the heat treatment temperature.

Finally, a layer of 50 nm ZnO was deposited above the temperature sensing layer as the isolation layer at a sputtering power of 120 W and pressure of 0.8 Pa. Figure 3b presents the schematic diagram of a cross section of the sensor. Figure 3c shows the flexible temperature sensor.

## 3. Results and Discussion

### 3.1. Static Characteristic Analysis

#### 3.1.1. Temperature Coefficient of Resistance

**Figure 4:**
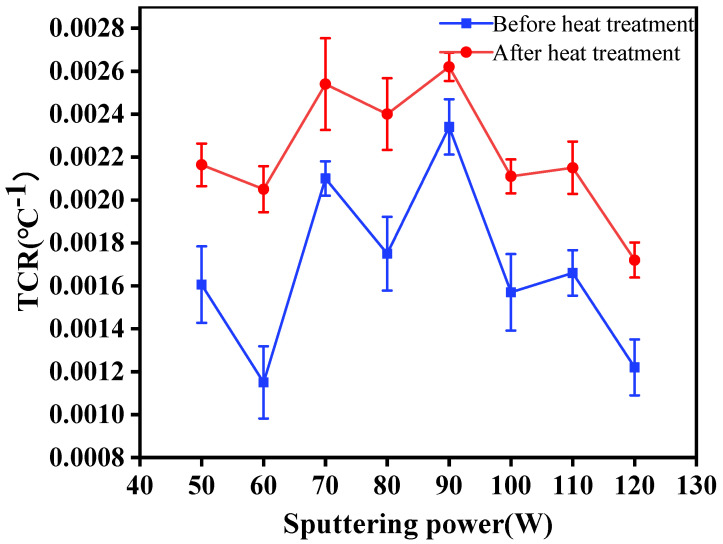
TCR of temperature sensing layers at specific sputtering powers.

The square resistance of Ag layers testing was carried out between 25 and 42 °C. The square resistance was measured five times repeatedly at specific sputtering powers, and the TCR was calculated and averaged. TCR was calculated using the following formula:

(1) $$\alpha \;=\frac{\Delta R}{R_{0}\Delta T}$$

where ΔR is the change of total resistance when the temperature changes between 25 °C and 42 °C, R_0_ is the initial resistance value of temperature sensing layers at 25 °C, ΔT is temperature difference, and α is TCR. Metal conductors are sensitive to temperature. When temperature rises, the vibration energy of atoms will increase in the metal, collision of free electrons will intensify, and resistance will increase, showing a positive TCR. The sensitivity of a thermal resistance temperature sensor is usually evaluated by TCR. The higher the TCR is, the higher the sensitivity. The relationship between averaged TCR and sputtering power were shown in Figure 4. It can be seen that TCR increased firstly and then decreased with the increment of power. After vacuum heat treatment at 160 °C, TCR of Ag layers made at specific powers increased obviously. Respectively, it was shown that sputtering power had a great influence on the conductivity of the Ag layers. When the sputtering power was low or high, the sensitivity of Ag layers would decrease, and the TCR would decrease. When the sputtering power was 90 W, the TCR of the temperature sensing layer was the highest, which was 0.00234 °C^−1^. After vacuum heat treatment, the change range of the square resistance of the temperature sensing layer increased, the sensitivity of sensing layer increased, and TCR increased. When sputtering power was 90 W, the TCR of the temperature sensing layer of the flexible temperature sensor was the highest, which was 0.00262 °C^−1^ after vacuum heat treatment, and the standard deviation of the measurement result was the smallest, which was 0.001544.

#### 3.1.2. Linearity Analysis

The linearity of a flexible temperature sensor is an essential characteristic to evaluate its properties. Linearity is the degree to which the input value of sensor is linear to output value. At ideal conditions, the input-output curves are linear and can be expressed by the following formula:

Y = AX + B,(2)

In the formula, Y represents the output value of resistance, X is temperature input value, while A and B are constants. The linearity of Ag layers was evaluated by fitting degree R^2^. The greater the value of R^2^, the better the linearity of the temperature sensitive layers. As shown in Table 1, only when sputtering power was 60 W and without heat treatment was R^2^ (0.9874) less than 0.99. The experiment results showed that sputtering power had little effect on the linearity of the flexible temperature sensor, and linearity can be maintained well at each power. After heat treatment, the linearity of Ag temperature sensing layers changed little with the increment of power and became stable.

#### 3.1.3. Hysteresis Analysis

**Figure 5:**
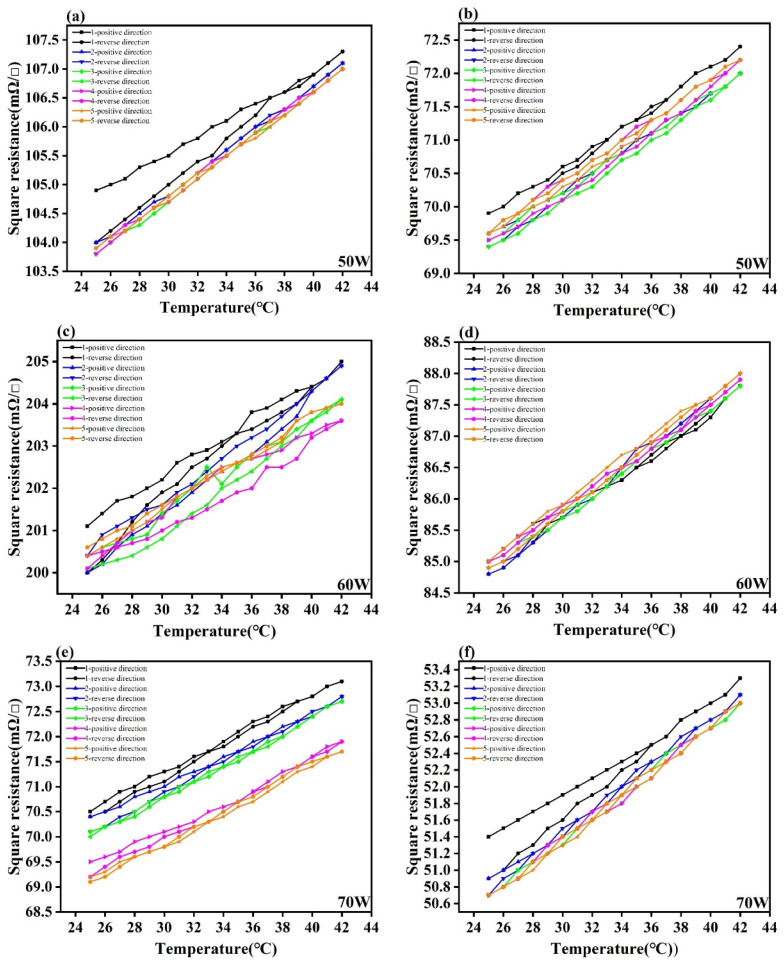

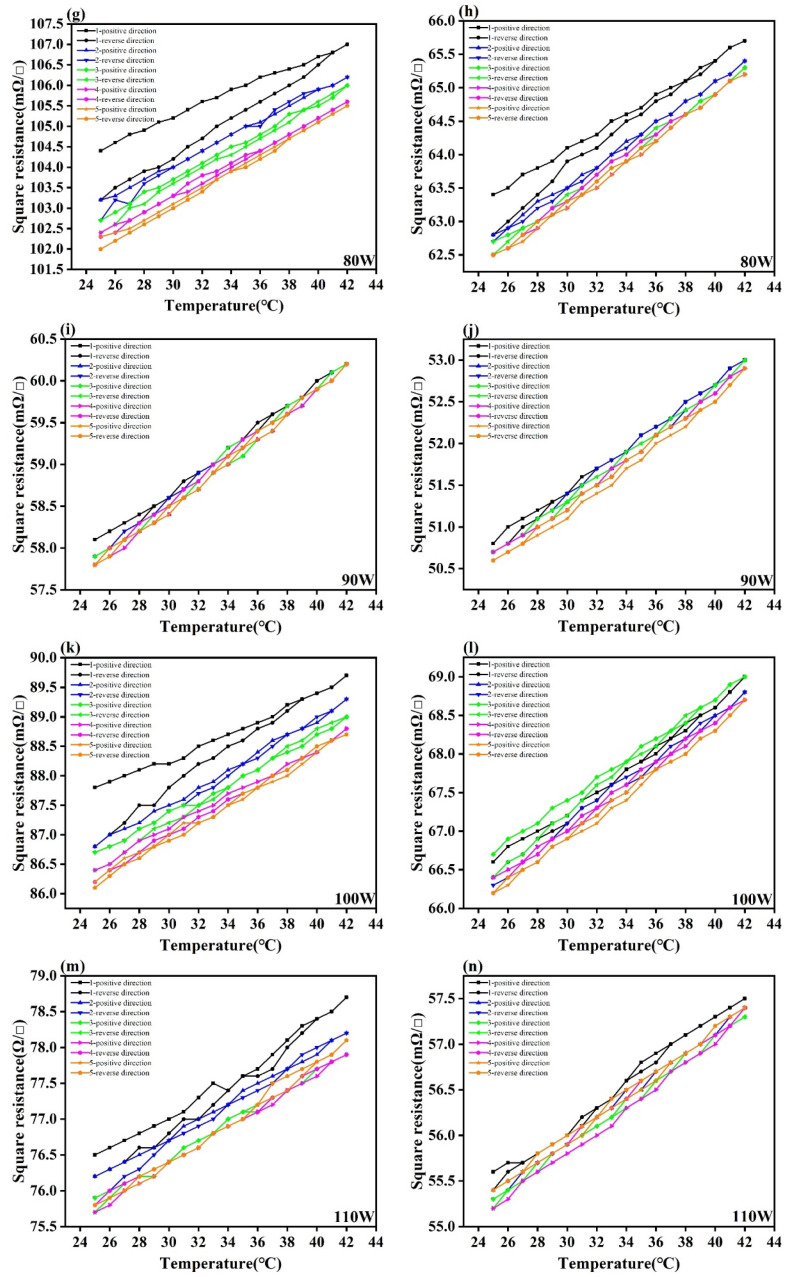

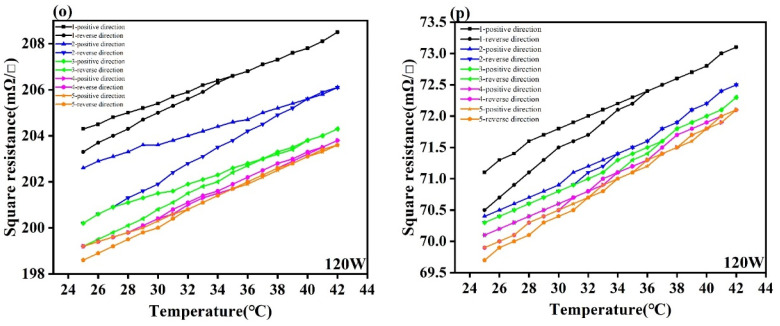
Left column—hysteresis curves at each power before heat treatment; right column—hysteresis curves at each power after heat treatment. (**a**) Hysteresis curves of Ag layer before heat treatment at 50 W; (**b**) Hysteresis curves of Ag layer after heat treatment at 50 W; (**c**) Hysteresis curves of Ag layer before heat treatment at 60 W; (**d**) Hysteresis curves of Ag layer after heat treatment at 60 W; (**e**) Hysteresis curves of Ag layer before heat treatment at 70 W; (**f**) Hysteresis curves of Ag layer after heat treatment at 70 W; (**g**) Hysteresis curves of Ag layer before heat treatment at 80 W; (**h**) Hysteresis curves of Ag layer after heat treatment at 80 W; (**i**) Hysteresis curves of Ag layer before heat treatment at 90 W; (**j**) Hysteresis curves of Ag layer after heat treatment at 90 W; (**k**) Hysteresis curves of Ag layer before heat treatment at 100 W; (**l**) Hysteresis curves of Ag layer after heat treatment at 100 W; (**m**) Hysteresis curves of Ag layer before heat treatment at 110 W; (**n**) Hysteresis curves of Ag layer after heat treatment at 110 W; (**o**) Hysteresis curves of Ag layer before heat treatment at 120W; (**p**) Hysteresis curves of Ag layer after heat treatment at 120 W.

Hysteresis is a performance index that characterizes the degree of misalignment of the input-output curves of a sensor during the temperature changes of forward and reverse strokes. The calculation formula is as follows:

E_max_ = (±∆m/Y_FS_) × 100% (3)

where ∆m is the maximum hysteresis difference generated within the entire measurement range and Y_FS_ is the full-scale output value. The smaller the hysteresis value, the higher the coincidence degree between the forward and reverse stroke curves of the sensor. As shown in Figure 5, the hysteresis values were different at different sputtering powers. When the sputtering power was 90 W, the hysteresis was the minimum, which was only 5.82%, and the standard deviation was the lowest, which was 2.46. After heat treatment at 160 °C, the hysteresis values of temperature sensing layers were reduced obviously and became stable relatively at most sputtering powers. The experimental results showed that sputtering power had a great influence on the hysteresis of temperature sensing layers. Moreover, heat treatment can also decrease the hysteresis of the temperature sensing layer and improve the coincidence degree of resistance in forward and reverse stroke.

#### 3.1.4. Drift

**Figure 6:**
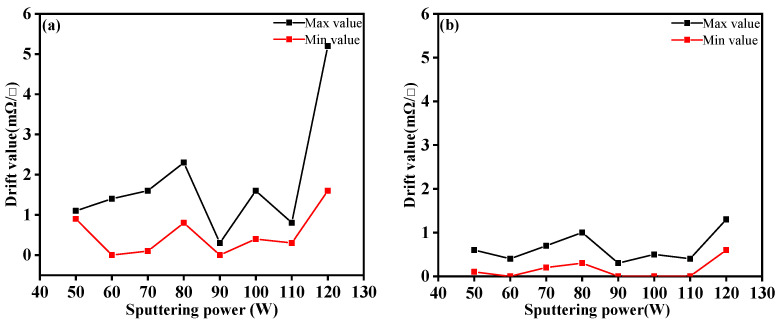
(**a**) Drift value of Ag layers before heat treatment at specific sputtering powers; (**b**) Drift value of Ag layers after heat treatment at specific sputtering powers.

Drift refers to the change of resistance of temperature sensors due to external interference, such as humidity, noise, air velocity, etc., at the condition that the temperature sensor input value is unchanged. Drift value is the difference of square resistance measured twice at the same temperature. Figure 6 presents the maximum and minimum drift curves of square resistance by five measurements for the Ag layers. It can be seen from the figure that sputtering power had a certain influence on the drift values of Ag layers. Without heat treatment, the drift value was up to 5.2 mΩ/□. After heat treatment, the maximum and minimum drift values of the temperature sensing layer were reduced obviously at each power. The experimental results showed that heat treatment can reduce the drift values and enhance the stability of the sensor. When the sputtering power was 90 W, the drift value was the smallest, within the range of 0~0.3 mΩ/□.

#### 3.1.5. Reliability

**Figure 7:**
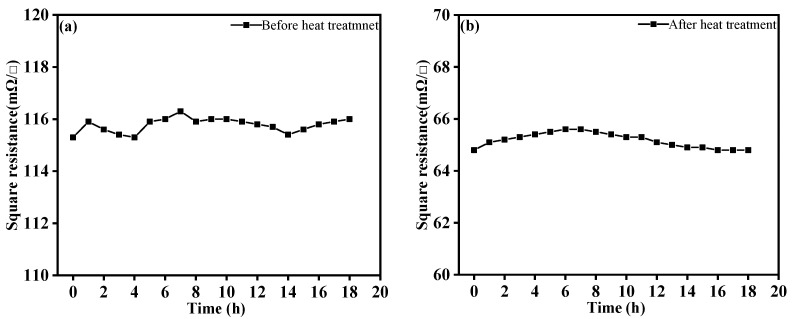
(**a**) Variation of square resistance of Ag layers with time at 30 °C before heat treatment (**b**) Variation of square resistance of Ag layers with time at 30 °C after heat treatment.

In this work, the reliability of Ag layers at 90 W with time and TCR after 200 bending times were tested. As shown Figure 7, the square resistance fluctuated by 1 mΩ before heat treatment, and its reliability was improved obviously after heat treatment within 18 h, showing good reliability. The reason for resistance fluctuation was that testing started in the morning and ended in the evening, so the change of ambient temperature will affect the measurement of the values of square resistance during this period. The values of the Ag layers increased first and then decreased. After 200 bending times tests, the TCR of the Ag layer was 0.002188 °C^−1^ and 0.00253 °C^−1^ before and after heat treatment, and the TCR changed by 6.5% and 3.4%, respectively. The test results showed that the change of ambient temperature will affect the resistance value of Ag layer. The TCR changed little after the bending test, and the reliability and bending resistance of the heat-treated temperature sensing layer were effectively improved.

### 3.2. Structure Analysis

**Figure 8:**
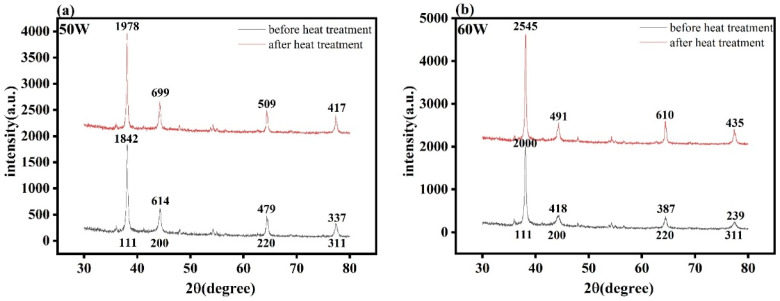

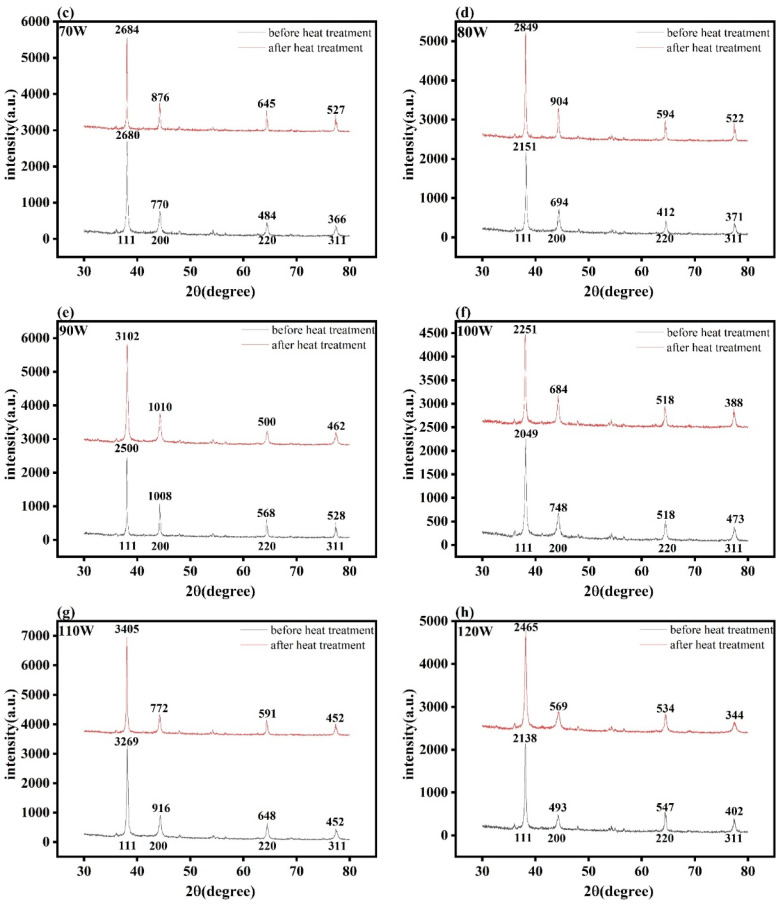
XRD figures of Ag layers before and after heat treatment. (**a**) XRD figure of Ag layers at 50 W; (**b**) XRD figure of Ag layers at 60 W; (**c**) XRD figure of Ag layers at 70 W; (**d**) XRD figure of Ag layers at 80 W; (**e**) XRD figure of Ag layers at 90 W; (**f**) XRD figure of Ag layers at 100 W; (**g**) XRD figure of Ag layers at 110 W; (**h**) XRD figure of Ag layers at 120 W.

Figure 8 shows the XRD test results of Ag temperature sensing layers at specific sputtering powers. It can be seen that there were four peaks with different intensities at 38.12, 44.28, 64.43, and 77.47, corresponding to the peaks of Ag(111), Ag(200), Ag(220), and Ag(311), respectively. After heat treatment, the intensity of the Ag(111) peak was increased significantly at every sputtering power, which can be attributed to the decrease of lattice defects of the Ag layers, such as vacancies, dislocations, interstitials, and grain boundaries [26,28], indicating the growth of grains being more perfect. The intensities of the other three peaks were also mostly increased after heat treatment. This meant that heat treatment would have a certain influence on the crystallization of the Ag layers. The TCR of the temperature sensing layers can be improved further.

**Figure 9:**
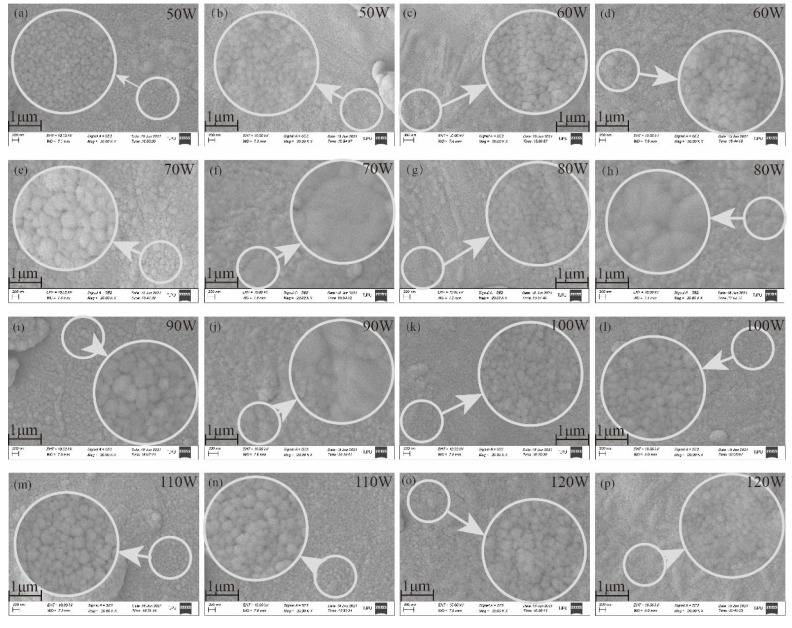
(**a**) SEM of Ag layer before heat treatment at 50 W; (**b**) SEM of Ag layer after heat treatment at 50 W; (**c**) SEM of Ag layer before heat treatment at 60 W; (**d**) SEM of Ag layer after heat treatment at 60 W; (**e**) SEM of Ag layer before heat treatment at 70 W; (**f**) SEM of Ag layer after heat treatment at 70 W; (**g**) SEM of Ag layer before heat treatment at 80 W; (**h**) SEM of Ag layer after heat treatment at 80 W; (**i**) SEM of Ag layer before heat treatment at 90 W; (**j**) SEM of Ag layer after heat treatment at 90 W; (**k**) SEM of Ag layer before heat treatment at 100 W; (**l**) SEM of Ag layer after heat treatment at 100 W; (**m**) SEM of Ag layer before heat treatment at 110 W; (**n**) SEM of Ag layer after heat treatment at 110 W; (**o**) SEM of Ag layer before heat treatment at 120W; (**p**) SEM of Ag layer after heat treatment at 120 W.

Figure 9 shows the SEM results of the temperature sensing layers of Ag at specific sputtering powers before and after heat treatment. It can be seen that when the sputtering powers were lower, the particles formed by Ag nanoparticles on the substrate were smaller and the TCR lower. Ag nanoparticles received less initial energy and lower kinetic energy at lower powers, and they were not tightly bonded on the substrate, resulting in smaller particles. The conductivity of the Ag temperature sensing layers was poor. When the temperature changed, the resistance changed in a small range and TCR decreased. As shown in Figure 9a, when the sputtering power was 50 W, the particles formed on the fabric substrate were smaller, and TCR was lower. After heat treatment, as shown in Figure 9b, as the internal stress of Ag layer was released, the Ag particles connected more compactly on the fabric substrate and the TCR became higher correspondingly. As shown in Figure 9c, when the sputtering power was 60 W, the Ag layer was uneven, and more cracks appeared, which led to the poor conductivity and decrease of TCR. After heat treatment, as shown in Figure 9d, the Ag particles became larger and the TCR was improved. As shown in Figure 9e–j, when sputtering powers were 70 W, 80 W, and 90 W, respectively, the particles formed by Ag nanoparticles on the fabric substrate were larger. After heat treatment, the Ag particles changed most obviously, and the TCR was also higher. As the sputtering powers were higher, the initial energy obtained by Ag nanoparticles was higher. The kinetic energy of nanoparticles was high when they flew from the target to the substrate. They bounced on the substrate surface. It is difficult for them to form an even and uniform layer, which leads to poor conductivity. When the temperature changed, the resistance changed in a small range and TCR decreased. As shown in Figure 9k–p, when the sputtering power was 100 W, the particles formed by Ag nanoparticles on the fabric substrate became smaller, the layer was of poor quality, and the TCR decreased greatly. When the sputtering power was 110 W, the particles formed by Ag nanoparticles became slightly larger, and the TCR increased slightly. When the sputtering power was 120 W, the layer became rough and uneven, and the TCR decreased significantly. The experimental results showed that improving the tightness between Ag particles, increasing the size of Ag particles, and releasing their internal stress will significantly improve the conductivity of the Ag layers, improving the TCR of the flexible temperature sensor further.

**Figure 10:**
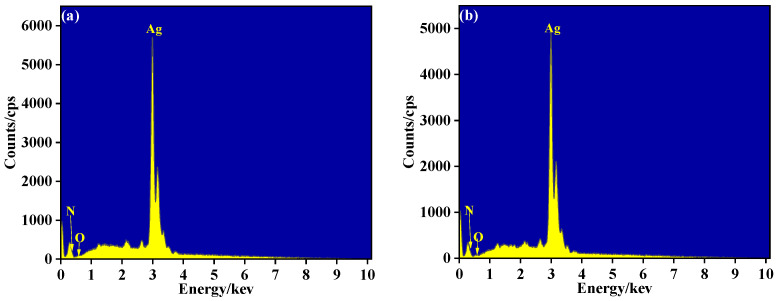
(**a**) EDS of Ag layer before heat treatment at 90 W; (**b**) EDS of Ag layer after heat treatment at 90 W.

Figure 10 shows EDS testing results of Ag temperature sensing layers at 90 W. The testing was carried out when the Ag layers was placed for a long time. It can be seen from Figure 10 that a large amount of Ag nanoparticles(98.88 wt.%) had been deposited on the surface of the substrate, and the layers contained a few O(0.5 wt.%) and N(0.62 wt.%) elements. After vacuum heat treatment, the contents of O(0.56 wt.%) and N(0.48 wt.%) hardly changed. The experimental results showed that the element content had almost no change before and after heat treatment. The temperature sensing layer has strong oxidation resistance.

### 3.3. Comparison of Sensing Properties

Films deposited on a textile substrate presented good uniformity and high adhesion by magnetron sputtering, and had the characteristics of low temperature deposition and high-speed sputtering [29]. In this paper, Ag layer flexible temperature sensor fabricated by depositing Ag nanoparticles on TPU coated polyester fabric had ultra-high conductivity by the sputtering method, which was measured to be between 3333 S/m and 10,000 S/m at specific sputtering powers, far higher than that of silicone fibers filled with Ag flakes (conductivity was 470 S/m), the nylon fiber mat (conductivity was 1800 S/m) [30], and AGNWS integrated into a stretchable AGNP/SBS [31]. This showed that the Ag temperature sensing layer deposited on the TPU coated polyester fabric had super conductivity. Its TCR was better than that of flexible temperature sensors fabricated by other methods, such as knit braided RTD (TCR = 2.24 × 10^−3^ °C^−1^), braided RTD (TCR = 1.53 × 10^−3^ °C^−1^), double covered RTD (TCR = 2.58 × 10^−3^ °C^−1^) [32], and RGO-Ag nanocomposite film integrated on the flexible Kapton sheets/membrane platform enabling a temperature sensor [33]. In the previous experimental exploration, most researchers have opted to embed rigid sensors into fabrics [34], or to use polymer materials such as colorless polyimide [35] as the substrate for flexible temperature sensors. Sensors fabricated by these two methods suffer from low softness and comfortableness. The innovation of this experiment was integrating the temperature sensing layer material onto the fabric. The flexible temperature sensor fabricated in this way not only had high comfortableness, but also high conductivity and TCR, which laid a solid foundation for real-time temperature monitoring in the medical and wearable fields.

## 4. Conclusions

In this paper, a novel flexible temperature sensor based on TPU-coated polyester fabric was fabricated. In the tested temperature range, the TCR increased first and then decreased with the increment of sputtering power. When the sputtering power was 90 W, TCR reached the highest value of 0.00234 °C^−1^. After heat treatment at 160 °C, TCR further increased to 0.00262 °C^−1^. X-ray diffractometry, thermal field-emission scanning electron microscopy, and energy dispersive spectrometry were used to characterize the structure and analyze the element content of Ag layers. It was found that the crystallinity of Ag particles can be improved, the bonding between Ag particles became closer, the sensitivity to temperature increased, the conductivity became better, and the TCR was improved after heat treatment at the same sputtering conditions. SEM testing results showed that the better the smoothness of the Ag layers and the larger the particles formed by Ag nanoparticles, the better the conductivity of the Ag layers and the higher the TCR. The EDS testing results showed that the element contents of O and N in the Ag layers have not changed before and after heat treatment, and the temperature sensing layer had antioxidant capacity. Thus, this research is helpful for temperature monitoring systems for smart wearable products.

## Figures and Tables

**Table 1:** Linearity of temperature sensing layers at specific powers.

Power (W)	50	60	70	80	90	100	110	120
before heat treatment								
average of R^2^	0.9985	0.9874	0.9950	0.9977	0.9959	0.9950	0.9945	0.9971
after heat treatment								
average of R^2^	0.9964	0.9965	0.9962	0.9973	0.9955	0.9966	0.9948	0.9951

## Data Availability

The data presented in this study are available on request from the corresponding author.
